# Large-Scale Tissue Microarray Evaluation Corroborates High Specificity of High-Level Arginase-1 Immunostaining for Hepatocellular Carcinoma

**DOI:** 10.3390/diagnostics11122351

**Published:** 2021-12-14

**Authors:** Maximilian Lennartz, Eva Gehrig, Sören Weidemann, Natalia Gorbokon, Anne Menz, Franziska Büscheck, Claudia Hube-Magg, Andrea Hinsch, Viktor Reiswich, Doris Höflmayer, Christoph Fraune, Frank Jacobsen, Christian Bernreuther, Patrick Lebok, Guido Sauter, Waldemar Wilczak, Stefan Steurer, Eike Burandt, Andreas H. Marx, Ronald Simon, Till Krech, Till S. Clauditz, Sarah Minner, David Dum, Ria Uhlig

**Affiliations:** 1 Institute of Pathology, University Medical Center Hamburg-Eppendorf, 20246 Hamburg, Germany; m.lennartz@uke.de (M.L.); eva.gehrig@hotmail.de (E.G.); s.weidemann@uke.de (S.W.); n.gorbokon@uke.de (N.G.); a.menz@uke.de (A.M.); f.buescheck@uke.de (F.B.); c.hube@uke.de (C.H.-M.); a.hinsch@uke.de (A.H.); v.reiswich@uke.de (V.R.); d.hoeflmayer@uke.de (D.H.); c.fraune@uke.de (C.F.); f.jacobsen@uke.de (F.J.); c.bernreuther@uke.de (C.B.); p.lebok@uke.de (P.L.); g.sauter@uke.de (G.S.); w.wilczak@uke.de (W.W.); s.steurer@uke.de (S.S.); e.burandt@uke.de (E.B.); andreas.marx@klinikum-fuerth.de (A.H.M.); t.krech@uke.de (T.K.); t.clauditz@uke.de (T.S.C.); s.minner@uke.de (S.M.); d.dum@uke.de (D.D.); r.uhlig@uke.de (R.U.); 2 Department of Pathology, Academic Hospital Fuerth, 90766 Fuerth, Germany; 3 Institute of Pathology, Clinical Center Osnabrueck, 49076 Osnabrueck, Germany

**Keywords:** arginase-1, immunohistochemistry, tissue micro array, neoplastic tissue, hepatocellular carcinoma

## Abstract

Arginase-1 catalyzes the conversion of arginine to ornithine and urea. Because of its predominant expression in hepatocytes, it serves as a marker for hepatocellular carcinoma, although other tumor entities can also express arginase-1. To comprehensively determine arginase-1 expression in normal and neoplastic tissues, tissue microarrays containing 14,912 samples from 117 different tumor types and 608 samples of 76 different normal tissue types were analyzed by immunohistochemistry. In normal tissues, arginase-1 was expressed in the liver, the granular layer of the epidermis, and in granulocytes. Among tumors, a nuclear and cytoplasmic arginase-1 immunostaining was predominantly observed in hepatocellular carcinoma, where 96% of 49 cancers were at least moderately positive. Although 22 additional tumor categories showed occasional arginase immunostaining, strong staining was exceedingly rare in these entities. Staining of a few tumor cells was observed in squamous cell carcinomas of various sites. Staining typically involved maturing cells with the beginning of keratinization in these tumors and was significantly associated with a low grade in 635 squamous cell carcinomas of various sites (*p* = 0.003). Teratoma, urothelial carcinoma and pleomorphic adenomas sometimes also showed arginase expression in areas with squamous differentiation. In summary, arginase-1 immunohistochemistry is highly sensitive and specific for hepatocellular carcinoma if weak and focal staining is disregarded.

## 1. Introduction

Arginase-1 is encoded by the ARG1 gene located at 6q23. It acts as a cytosolic manganese-dependent enzyme that catalyzes the conversion of arginine to ornithine and urea in the final step of the urea cycle [1,2,3,4]. Among normal tissues, it is predominantly expressed in hepatocytes and inflammatory cells. Because arginase-1 expression is usually retained in hepatocellular carcinoma, a cancer derived from hepatocytes, the immunohistochemical detection of arginase expression is commonly used to support the difficult distinction of hepatocellular carcinoma from cholangiocellular carcinoma and metastases to the liver [5]. This procedure is supported by more than 20 studies demonstrating arginase-1 expression in 80–100% of hepatocellular carcinomas [6,7,8,9,10,11,12,13,14,15,16,17,18,19,20,21,22,23,24,25,26].

Multiple studies have suggested that arginase-1 expression of tumor cells is largely absent in other important tumor types, such as renal cell carcinomas [8,25], ductal adenocarcinoma of the pancreas [8,21], gastric adenocarcinoma [25], esophageal adenocarcinoma [25], adenocarcinoma of the lung [25], and in lobular breast cancer [25]. The extent to which arginase-1 expression is specific for hepatocellular carcinoma is still unclear, however. Studies have demonstrated arginase-1 expression in 0–7% of prostate cancer [25,26,27], 6% of adenocarcinomas of the ampulla Vateri [28], 84% of squamous cell carcinoma of the oral cavity and the larynx [14], and “high” expression in 47% of the 79 analyzed invasive breast carcinomas of no special type (NST) [29]. In one study, multiple soft tissue tumors were described to show arginase-1 expression in up to 100% of cases [30]. The published arginase-1 positivity rates are also markedly variable in tumor entities that are often seen in the liver such as cholangiocarcinoma (positivity described in 0–68% of cases, [8,19,24,25,26,31], breast cancer NST (0–47%), [8,18,26,29]), colorectal adenocarcinoma (0–100%) [8,26,32,33] and even hepatocellular carcinoma 45–100%, [6,8,9,11,12,22,26]. This data variability is most likely due to the use of different antibodies, staining protocols and criteria for staining interpretation in the respective studies.

To better understand the prevalence and diagnostic utility of arginase-1 expression in cancer, a comprehensive study analyzing large numbers of neoplastic and non-neoplastic tissues under highly standardized conditions is desirable. For this purpose, arginase-1 expression was analyzed in more than 14,000 tumor tissue samples from 117 different tumor types and subtypes as well as 76 non-neoplastic tissue categories by immunohistochemistry (IHC) in a tissue microarray (TMA) format in this study.

## 2. Materials and Methods

Tissue Microarrays (TMAs). Our normal tissue TMA was composed of 8 samples from 8 different donors for each of the 76 different normal tissue types (608 samples on one slide). The cancer TMAs contained a total of 14,912 primary tumors from 117 tumor types and subtypes. The composition of normal and tumor TMAs is described in the results section. All samples were obtained from the archives of the Institutes of Pathology, University Hospital of Hamburg, Germany, the Institute of Pathology, Clinical Center Osnabrueck, Germany, and Department of Pathology, Academic Hospital Fuerth, Germany. Tissues were fixed in 4% buffered formalin and then embedded in paraffin. The TMA manufacturing process was described earlier in detail [34,35,36]. In brief, one tissue spot (diameter: 0.6 mm) was transferred from a cancer containing donor block to an empty recipient paraffin block. The use of archived remnants of diagnostic tissues for TMA manufacturing, their analysis for research purposes, and patient data were conducted according to local laws (HmbKHG, §12) and the analysis had been approved by the local ethics committee (Ethics commission Hamburg, WF-049/09). All work has been carried out in compliance with the Helsinki Declaration.

Immunohistochemistry (IHC). Freshly cut TMA sections were all immunostained on one day and in one experiment. Slides were deparaffinized and exposed to heat-induced antigen retrieval for 5 min in an autoclave at 121 °C in a pH 7.8 TRIS-EDTA-citrate buffer. The primary antibody specific to arginase-1 (rabbit recombinant, MSVA-511R, MS Validated Antibodies, GmbH, Hamburg, Germany) was applied at 37 °C for 60 min at a dilution of 1: 150 in antibody diluent from Agilent/Dako #S080938. The bound antibody was then visualized using the EnVision Kit (Agilent/Dako #K5007) according to the manufacturer’s directions. For tumor tissues, the percentage of positive neoplastic cells was estimated, and the staining intensity was semi-quantitatively recorded (0, 1+, 2+, 3+). For statistical analyses, the staining results were categorized into four groups. Tumors without any staining were considered negative. Tumors with 1+ staining intensity in ≤70% of cells or 2+ intensity in ≤30% of cells were considered weakly positive. Tumors with 1+ staining intensity in >70% of cells, 2+ intensity in 31–70%, or 3+ intensity in ≤30% were considered moderately positive. Tumors with 2+ intensity in >70% or 3+ intensity in >30% of cells were considered strongly positive. For antibody validation, the normal tissue TMA was also stained with a second anti-arginase-1 antibody (Cell Marque clone SP156, Cat. # 380R-18) for 20 min, at a dilution of 1:6.25 in a Dako Link48 autostainer after Flex-high antigen retrieval. 

Statistics. Statistical calculations were performed with JMP 14 software (SAS Institute Inc., Cary, NC 27513, USA). The chi²-test was performed to search for associations between arginase immunostaining and a tumor phenotype in squamous cell cancers.

## 3. Results

### 3.1. Technical Issues

A total of 12,047 (81%) of 14,912 tumor samples were interpretable in our TMA analysis. Non-interpretable samples demonstrated a lack of unequivocal tumor cells or loss of tissue location during technical procedures. Sufficient numbers of samples of each normal tissue type were evaluable.

### 3.2. Staining Pattern in Normal Tissues

**Figure 1:**
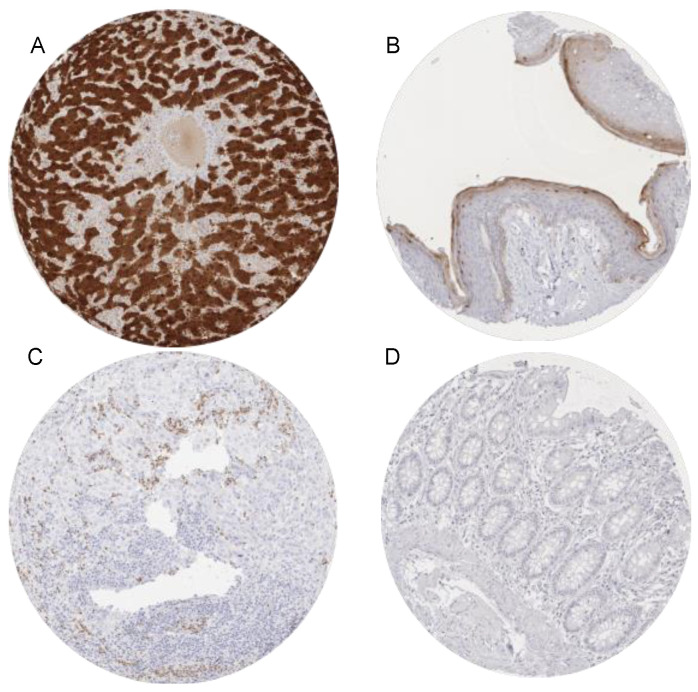
Arginase-1 immunostaining in normal cells. The panels show a strong cytoplasmic and nuclear arginase-1 positivity of hepatocytes (**A**). Hepatocyte staining is strong enough that some staining is also seen in the adjacent stroma (contamination artifact). A weak to moderate cytoplasmic and nuclear arginase-1 immunostaining occurs in the granular cell layer of the keratinizing squamous epithelium of the skin (**B**) while staining is weak and cytoplasmic in granulocytes infiltrating an arginase-1 negative cholangiocellular carcinoma (**C**). Arginase-1 immunostaining is absent in colon epithelium (**D**). Magnification 100×, TMA spot size 600 µm.

The Arginase-1 immunostaining was typically cytoplasmic and nuclear. By far the strongest Arginase-1 immunostaining was seen in hepatocytes. Moderate to strong cytoplasmic and nuclear arginase immunostaining also occurred in the granular cell layer of keratinizing squamous epithelium of the skin. Moderate staining occurred in granulocytes and its precursor cells in the bone marrow. A weak to moderate Arginase-1 positivity was seen in a fraction of the decidua cells. Representative images are shown in Figure 1. Arginase-1 immunostaining was not observed in any other epithelial cells from the gastrointestinal tract, urothelium, non-keratinizing squamous epithelia, pancreas, salivary glands, thyroid, parathyroid gland, adenohypophysis, adrenal gland, prostate, epididymis, testis, seminal vesicle, endometrium, endocervix, fallopian tube, kidney, respiratory epithelium, lung, placenta, various types of muscle cells, myometrium, lymphatic organs, endothelium, brain and neurohypophysis. All positive stainings were also confirmed by the use of a second independent antibody (Cell Marque clone SP156, Supplementary Materials Figure S1).

### 3.3. Arginase in Cancer

Arginase expression was predominantly observed in hepatocellular carcinoma, where 88% of 49 tumors showed a strong arginase positivity and 96% a moderate arginase positivity independently from the tumor stage (*p* = 0.4132). A characteristic nuclear and cytoplasmic staining was typically seen in these tumors. Although 22 additional tumor categories showed arginase immunostaining in a much smaller fraction of cases, strong and even moderate arginase-1 staining was exceedingly rare in these entities (Table 1). 

The focal staining of a few tumor cells was observed in squamous cell carcinomas for various sites, where it was significantly associated with a low histological tumor grade (*p* = 0.003, Table 2). 

Arginase-1 staining was unrelated to HPV-status, however (Table 3). 

**Figure 2:**
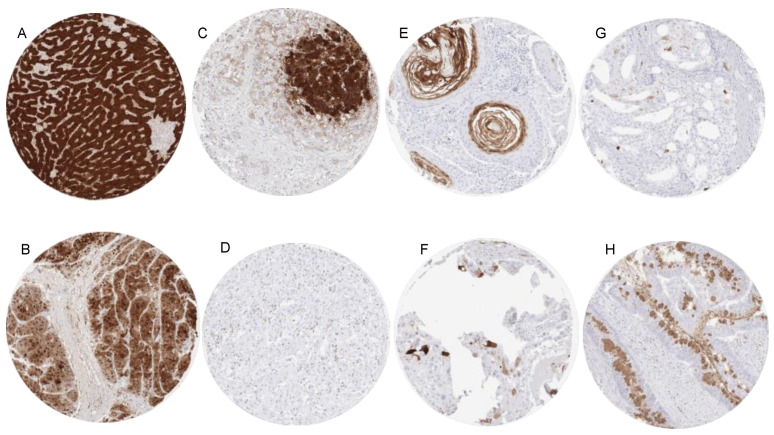
Arginase-1 immunostaining in cancer. The panels show examples of a strong (**A**) and a more variable, moderate to strong (**B**) nuclear and cytoplasmic arginase-1 staining in hepatocellular carcinomas. A weak and purely cytoplasmic arginase-1 staining in a cholangiocellular carcinoma (**C**) which is particularly seen in cells adjacent to strongly positive normal hepatocytes may reflect a “contamination” artifact. In another cholangiocellular carcinoma, tumor cells are arginase-1 negative, and staining is limited to tumor-associated granulocytes (**D**). A focal arginase-1 immunostaining is seen in keratinizing cells of a pharyngeal squamous cell carcinoma (**E**), a clear cell carcinoma of the ovary (**F**), and a Gleason 4 + 4 = 8 adenocarcinoma of the prostate (**G**). In a colorectal adenocarcinoma arginase-1 staining occurs in goblet cells and tumor-associated mucins (**H**). Magnification 100×, TMA spot size 600 µm.

**Figure 3:**
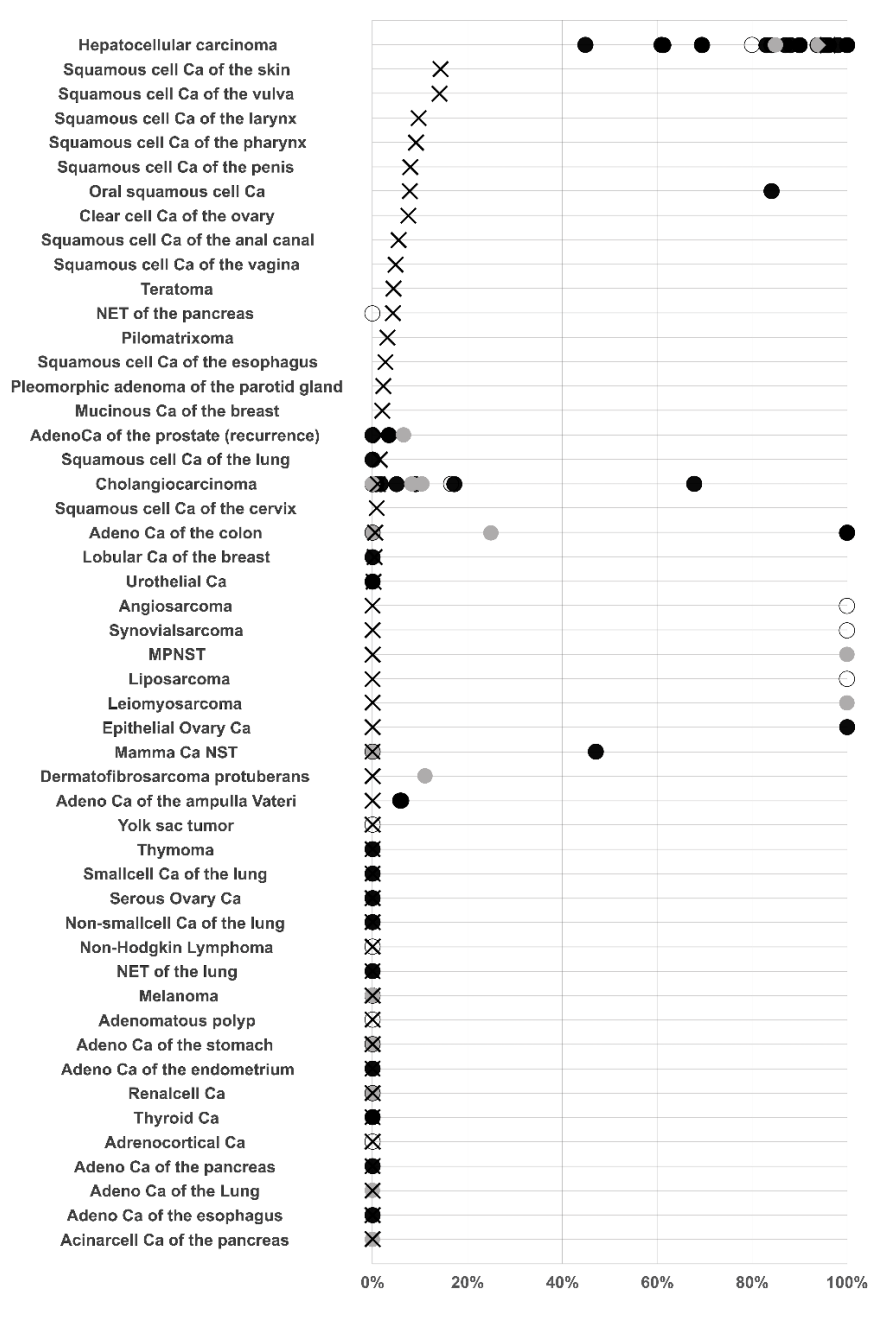
Graphical comparison of Arginase data from this study (x) in comparison with the previous literature (dots). Open: *n* = 1–10, grey: *n* = 11–25, black: *n* > 25. For comparison purposes, studies that did not differentiate between different tumor subtypes were marked with black dots and the overall positivity rate was applied to the different tumor subtypes present in our tumor microarrays. All studies are referred to in the reference list.

Similarly to arginase expression of normal squamous epithelium, arginase positivity typically involved maturing cells at the beginning of keratinization in these tumors. Rare positive cases of teratoma, urothelial carcinoma and pleomorphic adenomas also showed arginase expression in areas with squamous differentiation. Other tumor entities with occasional and mostly low-level arginase-1 immunostaining included clear cell carcinomas of the ovary, neuroendocrine tumors of the pancreas, mucinous and lobular carcinoma of the breast, cholangiocarcinoma, and colorectal adenocarcinoma. In rare case of arginase positive colorectal and mucinous breast carcinomas, arginase staining predominated in the intratumoral mucus (breast) or mucin producing goblet cells (colon). Representative images of arginase immunostaining in cancers are presented in Figure 2. A ranking order of arginase-1 positive and strongly positive cases in combination, with a summary of data from comparable studies, is provided in Figure 3.

## 4. Discussion

Given the large scale of our study, we placed emphasis on the thorough validation of our assay. The International Working Group for Antibody Validation (IWGAV) proposed that antibody validation for immunohistochemistry on formalin fixed tissues should include either a comparison of the findings obtained by two different independent antibodies, or a comparison with expression data obtained by another independent method [37]. To ensure that any antibody cross reactivity would be detected in our validation experiment, a broad range of different normal tissue categories were included in the analysis and the immunohistochemical staining results were not only compared with a second independent antibody, but also with RNA expression data derived from three independent RNA screening studies, including the Human Protein Atlas (HPA) RNA-seq tissue dataset [38], the FANTOM5 project [39,40], and the Genotype-Tissue Expression (GTEx) project [41]. The 76 different normal tissues that were selected for this experiment are likely to contain the majority of proteins occurring in the cells of adult humans. Therefore, we consider it likely that undesired antibody cross-reactivity can be detected with high certainty. A specific antibody reactivity in our experimental set-up is supported by the detection of significant arginase-1 immunostaining in all organs, with documented arginase-1 RNA expression (liver, skin, bone marrow, and granulocytes). The fact that RNA expression had not previously been documented for decidua cells is not surprising, given the paucity of these cells in mature placenta tissue samples that were systematically screened for RNA expression. True arginase-1 expression in decidua cells is, however, supported by the comparison with the independent antibody Cell Marque clone SP156, which also confirmed liver, skin, bone marrow and granulocyte staining (Supplementary Materials Figure S1).

A successful analysis of 12,047 cancers from 117 different tumor entities revealed a pattern of expression for arginase-1 that strongly correlated with the findings in normal tissues. Additionally, Arginase-1 expression was commonly seen in hepatocellular carcinomas, maturing/keratinizing zones of squamous cell carcinomas and in tumor infiltrating granulocytes. These findings greatly support the use of using arginase-1 immunohistochemistry for corroborating a suspected diagnosis of hepatocellular carcinoma. That 88% of 49 successfully analyzed HCCs showed a strong arginase-1 immunostaining and 96% showed a moderate staining fits well with earlier data. Of the 21 studies analyzing arginase-1 in hepatocellular carcinoma 9 described positivity rates of >90% [10,13,16,17,18,19,23,25]. That some studies also reported arginase-1 positivity rates of 44.9% [22], 80% [15] and 84% [14] reflects the inherent issue of incomplete reproducibility of immunostaining as long as the associated reagents and protocols are not standardized. Most of the previous studies agree that the few arginase-1 negative hepatocellular carcinomas are often poorly differentiated [8,12,22,26].

It is of note that the experimental set-up of this study resulted in some diffuse weak to moderate staining of stroma as well as of tumor tissue adjacent to strongly arginase-1 positive normal liver cells. This staining is likely to represent a contamination artifact due to the potential diffusion of abundant arginase-1 from the hepatocytes to adjacent tissue. Such a diffusion of abundant proteins may be facilitated by tissue damage, which can, for example, be caused by prolonged tissue ischemia before fixation occurs. Comparable artifacts occur, for example, in the thyroid, where some thyroglobulin immunostaining of medullary carcinomas can be observed in areas adjacent to normal follicles that contain abundant thyroglobulin [42]. The one arginase-1 positive cholangiocellular carcinoma of the liver showed a weak cytoplasmic staining for approximately 25% of the cells, which we accepted as “arginase-1 positive” because of the absence of strongly positive liver cells in this sample. If we had disregarded a weak to moderate arginase-1 immunostaining limited to the cytoplasm (non-nuclear) in samples from the liver, we would not have recorded any arginase-1 positivity in cholangiocellular carcinomas. In addition, our study did not reveal signs of intratumoral heterogeneity of arginase staining, although this aspect was not systematically addressed. It seems possible that a variable interpretation of such findings has contributed to the high variability of published data on arginase-1 positivity in cholangiocarcinoma, ranging from 0% [8] to 68% [31]. The pattern of arginase-1 immunostaining in squamous cell carcinomas closely resembled the findings in the normal keratinizing squamous epithelium. Arginase-1 positivity is focal in squamous cell carcinomas and tightly linked to a distinct maturation stage of the epithelium which is comparable to the granular layer of the normal squamous epithelium. As keratinization represents a feature of more mature squamous epithelium, it is not surprising that arginase-1 positivity was statistically linked to well-differentiated squamous cell carcinomas in this study. 

Arginase-1 positivity was only very rarely observed in non-hepatocellular, non-squamous cell carcinomas. In some of these cases, arginase-1 positivity occurred in areas of squamous differentiation (urothelial carcinoma, pleomorphic adenoma) or in the keratinizing squamous epithelium of a testicular teratoma. That a focal—mostly weak—arginase-1 immunostaining could also occasionally be seen in mucus producing cells of colorectal adenocarcinomas, clear cell carcinoma of the ovary, mucinous and lobular breast cancer may reflect the fact that genes without a cancer promoting function may be randomly activated in cancer cells [43,44].

In summary, these data show that strong nuclear and cytoplasmic arginase-1 immunostaining is largely specific for tumors of hepatocellular origin. The most significant issue to consider from a diagnostic point of view is the possibility of a contamination artifact in non-arginase-1 expressing tumor cells, adjacent to strongly arginase-1 positive normal liver cells. Furthermore, Arginase-1 positivity is also common in squamous cell carcinomas but limited to areas at the beginning of keratinization in these tumors.

## Figures and Tables

**Table 1:** Arginase-1 immunostaining in human tumors.

			Arginase-1 Immunostaining				
	Tumor Entity	On TMA (n)	Analyzable (n)	Negative (%)	Weak (%)	Moderate (%)	Strong (%)
Tumors of the skin	Pilomatrixoma	35	33	97.0	3.0	0.0	0.0
	Basal cell carcinoma	88	50	100.0	0.0	0.0	0.0
	Benign nevus	29	26	100.0	0.0	0.0	0.0
	Squamous cell carcinoma of the skin	90	77	85.7	13.0	1.3	0.0
	Malignant melanoma	48	43	100.0	0.0	0.0	0.0
	Merkel cell carcinoma	46	41	100.0	0.0	0.0	0.0
Tumors of the head and neck	Squamous cell carcinoma of the larynx	110	93	90.3	5.4	4.3	0.0
	Squamous cell carcinoma of the pharynx	60	44	90.9	9.1	0.0	0.0
	Oral squamous cell carcinoma (floor of the mouth)	130	115	92.2	7.0	0.9	0.0
	Pleomorphic adenoma of the parotid gland	50	45	97.8	2.2	0.0	0.0
	Warthin tumor of the parotid gland	49	49	100.0	0.0	0.0	0.0
	Basal cell adenoma of the salivary gland	15	14	100.0	0.0	0.0	0.0
Tumors of the lung, pleura and thymus	Adenocarcinoma of the lung	196	169	100.0	0.0	0.0	0.0
	Squamous cell carcinoma of the lung	80	68	98.5	1.5	0.0	0.0
	Small cell carcinoma of the lung	16	16	100.0	0.0	0.0	0.0
	Mesothelioma, epithelioid	39	33	100.0	0.0	0.0	0.0
	Mesothelioma, other types	76	63	100.0	0.0	0.0	0.0
	Thymoma	29	29	100.0	0.0	0.0	0.0
Tumors of the female genital tract	Squamous cell carcinoma of the vagina	78	63	95.2	4.8	0.0	0.0
	Squamous cell carcinoma of the vulva	130	114	86.0	13.2	0.9	0.0
	Squamous cell carcinoma of the cervix	129	119	99.2	0.8	0.0	0.0
	Endometrioid endometrial carcinoma	236	222	100.0	0.0	0.0	0.0
	Endometrial serous carcinoma	82	73	100.0	0.0	0.0	0.0
	Carcinosarcoma of the uterus	48	41	100.0	0.0	0.0	0.0
	Endometrial carcinoma, high grade, G3	13	13	100.0	0.0	0.0	0.0
	Endometrial clear cell carcinoma	8	7	100.0	0.0	0.0	0.0
	Endometrioid carcinoma of the ovary	110	90	100.0	0.0	0.0	0.0
	Serous carcinoma of the ovary	559	455	100.0	0.0	0.0	0.0
	Mucinous carcinoma of the ovary	96	74	100.0	0.0	0.0	0.0
	Clear cell carcinoma of the ovary	50	40	92.5	7.5	0.0	0.0
	Carcinosarcoma of the ovary	47	39	100.0	0.0	0.0	0.0
	Brenner tumor	9	9	100.0	0.0	0.0	0.0
Tumors of the breast	Invasive breast carcinoma of no special type	1345	1208	100.0	0.0	0.0	0.0
	Lobular carcinoma of the breast	293	252	99.6	0.4	0.0	0.0
	Medullary carcinoma of the breast	26	26	100.0	0.0	0.0	0.0
	Tubular carcinoma of the breast	27	26	100.0	0.0	0.0	0.0
	Mucinous carcinoma of the breast	58	49	98.0	2.0	0.0	0.0
	Phyllodes tumor of the breast	50	50	100.0	0.0	0.0	0.0
Tumors of the digestive system	Adenomatous polyp, low-grade dysplasia	50	48	100.0	0.0	0.0	0.0
	Adenomatous polyp, high-grade dysplasia	50	48	100.0	0.0	0.0	0.0
	Adenocarcinoma of the colon	1882	1610	99.5	0.3	0.1	0.1
	Gastric adenocarcinoma, diffuse type	176	142	100.0	0.0	0.0	0.0
	Gastric adenocarcinoma, intestinal type	174	132	100.0	0.0	0.0	0.0
	Gastric adenocarcinoma, mixed type	62	52	100.0	0.0	0.0	0.0
	Adenocarcinoma of the esophagus	83	61	100.0	0.0	0.0	0.0
	Squamous cell carcinoma of the esophagus	75	38	97.4	2.6	0.0	0.0
	Squamous cell carcinoma of the anal canal	89	73	94.5	4.1	1.4	0.0
	Cholangiocarcinoma	113	103	99.0	1.0	0.0	0.0
	Hepatocellular carcinoma	50	49	4.1	0.0	8.2	87.8
	Ductal adenocarcinoma of the pancreas	612	470	100.0	0.0	0.0	0.0
	Pancreatic/Ampullary adenocarcinoma	89	77	100.0	0.0	0.0	0.0
	Acinar cell carcinoma of the pancreas	16	14	100.0	0.0	0.0	0.0
	Gastrointestinal stromal tumor (GIST)	50	49	100.0	0.0	0.0	0.0
Tumors of the urinary system	Urothelial carcinoma, pT2-4 G3	1206	588	99.8	0.2	0.0	0.0
	Small cell neuroendocrine carcinoma of the bladder	20	19	100.0	0.0	0.0	0.0
	Sarcomatoid urothelial carcinoma	25	24	100.0	0.0	0.0	0.0
	Clear cell renal cell carcinoma	857	644	100.0	0.0	0.0	0.0
	Papillary renal cell carcinoma	255	185	100.0	0.0	0.0	0.0
	Clear cell (tubulo) papillary renal cell carcinoma	21	16	100.0	0.0	0.0	0.0
	Chromophobe renal cell carcinoma	131	107	100.0	0.0	0.0	0.0
	Oncocytoma	177	130	100.0	0.0	0.0	0.0
Tumors of the male genital organs	Adenocarcinoma of the prostate, Gleason 3 + 3	83	80	100.0	0.0	0.0	0.0
	Adenocarcinoma of the prostate, Gleason 4 + 4	80	72	100.0	0.0	0.0	0.0
	Adenocarcinoma of the prostate, Gleason 5 + 5	85	78	100.0	0.0	0.0	0.0
	Adenocarcinoma of the prostate (recurrence)	258	211	98.1	1.4	0.5	0.0
	Small cell neuroendocrine carcinoma of the prostate	19	17	100.0	0.0	0.0	0.0
	Seminoma	621	446	100.0	0.0	0.0	0.0
	Embryonal carcinoma of the testis	50	35	100.0	0.0	0.0	0.0
	Yolk sac tumor	50	31	100.0	0.0	0.0	0.0
	Teratoma	50	46	95.7	4.3	0.0	0.0
	Squamous cell carcinoma of the penis	80	63	92.1	7.9	0.0	0.0
Tumors of endocrine organs	Adenoma of the thyroid gland	114	104	100.0	0.0	0.0	0.0
	Papillary thyroid carcinoma	392	351	100.0	0.0	0.0	0.0
	Follicular thyroid carcinoma	154	136	100.0	0.0	0.0	0.0
	Medullary thyroid carcinoma	111	100	100.0	0.0	0.0	0.0
	Anaplastic thyroid carcinoma	45	43	100.0	0.0	0.0	0.0
	Adrenal cortical adenoma	50	21	100.0	0.0	0.0	0.0
	Adrenal cortical carcinoma	26	26	100.0	0.0	0.0	0.0
	Phaeochromocytoma	50	50	100.0	0.0	0.0	0.0
	Appendix, neuroendocrine tumor (NET)	22	13	100.0	0.0	0.0	0.0
	Colorectal, neuroendocrine tumor (NET)	12	11	100.0	0.0	0.0	0.0
	Ileum, neuroendocrine tumor (NET)	49	45	100.0	0.0	0.0	0.0
	Lung, neuroendocrine tumor (NET)	19	17	100.0	0.0	0.0	0.0
	Pancreas, neuroendocrine tumor (NET)	97	93	95.7	2.2	2.2	0.0
	Colorectal, neuroendocrine carcinoma (NEC)	12	10	100.0	0.0	0.0	0.0
	Gallbladder, neuroendocrine carcinoma (NEC)	4	4	100.0	0.0	0.0	0.0
	Pancreas, neuroendocrine carcinoma (NEC)	14	14	100.0	0.0	0.0	0.0
Tumors of haematopoietic and lymphoid tissues	Hodgkin Lymphoma	103	72	100.0	0.0	0.0	0.0
	Small lymphocytic lymphoma, B-cell type (B-SLL/B-CLL)	50	29	100.0	0.0	0.0	0.0
	Diffuse large B cell lymphoma (DLBCL)	114	95	100.0	0.0	0.0	0.0
	Follicular lymphoma	88	63	100.0	0.0	0.0	0.0
	T-cell Non Hodgkin lymphoma	24	14	100.0	0.0	0.0	0.0
	Mantle cell lymphoma	18	14	100.0	0.0	0.0	0.0
	Marginal zone lymphoma	16	10	100.0	0.0	0.0	0.0
	Diffuse large B-cell lymphoma (DLBCL) in the testis	16	13	100.0	0.0	0.0	0.0
	Burkitt lymphoma	5	1	100.0	0.0	0.0	0.0
Tumors of soft tissue and bone	Tenosynovial giant cell tumor	45	43	100.0	0.0	0.0	0.0
	Granular cell tumor	53	42	100.0	0.0	0.0	0.0
	Leiomyoma	50	48	100.0	0.0	0.0	0.0
	Leiomyosarcoma	87	81	100.0	0.0	0.0	0.0
	Liposarcoma	132	123	100.0	0.0	0.0	0.0
	Malignant peripheral nerve sheath tumor (MPNST)	13	12	100.0	0.0	0.0	0.0
	Myofibrosarcoma	26	26	100.0	0.0	0.0	0.0
	Angiosarcoma	73	61	100.0	0.0	0.0	0.0
	Angiomyolipoma	91	91	100.0	0.0	0.0	0.0
	Dermatofibrosarcoma protuberans	21	18	100.0	0.0	0.0	0.0
	Ganglioneuroma	14	13	100.0	0.0	0.0	0.0
	Kaposi sarcoma	8	6	100.0	0.0	0.0	0.0
	Neurofibroma	117	93	100.0	0.0	0.0	0.0
	Sarcoma, not otherwise specified (NOS)	74	71	100.0	0.0	0.0	0.0
	Paraganglioma	41	37	100.0	0.0	0.0	0.0
	Ewing sarcoma	23	18	100.0	0.0	0.0	0.0
	Rhabdomyosarcoma	6	6	100.0	0.0	0.0	0.0
	Schwannoma	121	103	100.0	0.0	0.0	0.0
	Synovial sarcoma	12	11	100.0	0.0	0.0	0.0
	Osteosarcoma	43	35	100.0	0.0	0.0	0.0
	Chondrosarcoma	38	24	100.0	0.0	0.0	0.0

**Table 2:** Arginase-1 immunostaining and tumor phenotype in 635 squamous cell carcinomas (SQCC) of various origins, including SQCC of the floor of the mouth (*n* = 99), pharynx (*n* = 40), larynx (*n* = 86), cervix (*n* = 116), vagina (*n* = 36), vulva (*n* = 106), penis (*n* = 59), skin (*n* = 45), and anal canal (*n* = 48).

	Arginase Immunostaining in SQCC					
	n	Neg. (%)	Weak (%)	Mod. (%)	Strong (%)	*p*
pT1	220	93.2	6.4	0.5	0.0	0.0761
pT2	221	94.6	5.4	0.0	0.0	
pT3	81	88.9	7.4	3.7	0.0	
pT4	113	91.2	6.2	2.7	0.0	
pN0	236	91.9	6.4	1.7	0.0	0.6054
pN+	233	94.0	5.2	0.9	0.0	
G1	28	85.7	10.7	3.6	0.0	0.0025
G2	340	90.3	8.5	1.2	0.0	
G3	226	97.8	1.8	0.4	0.0	

**Table 3:** Arginase-1 immunostaining and HPV status in squamous cell carcinomas.

	HPV Status	n	Arginase Status (%)				*p*
			Negative	Weak	Moderate	Strong	
All squamous cell cancers	negativ	250	90.0	8.4	1.6	0.0	0.7690
	positive	204	91.7	7.4	1.0	0.0	
Oral squamous cell carcinoma	negativ	56	89.3	8.9	1.8	0.0	0.8193
	positive	12	91.7	8.3	0.0	0.0	
Squamous cell carcinoma of the pharynx	negativ	18	94.4	5.6	0.0	0.0	0.4354
	positive	24	87.5	12.5	0.0	0.0	
Squamous cell carcinoma of the larynx	negativ	39	89.7	5.1	5.1	0.0	0.5278
	positive	7	85.7	14.3	0.0	0.0	
Squamous cell carcinoma of the cervix	negativ	10	100.0	0.0	0.0	0.0	-
	positive	64	100.0	0.0	0.0	0.0	
Squamous cell carcinoma of the vagina	negativ	15	93.3	6.7	0.0	0.0	0.2578
	positive	13	100.0	0.0	0.0	0.0	
Squamous cell carcinoma of the vulva	negativ	47	87.2	12.8	0.0	0.0	0.1330
	positive	24	70.8	25.0	4.2	0.0	
Squamous cell carcinoma of the penis	negativ	26	96.2	3.8	0.0	0.0	0.2657
	positive	35	88.6	11.4	0.0	0.0	
Squamous cell carcinoma of the skin	negativ	34	85.3	11.8	2.9	0.0	0.8551
	positive	1	100.0	0.0	0.0	0.0	
Squamous cell carcinoma of the anal canal	negativ	5	80.0	20.0	0.0	0.0	0.1347
	positive	24	95.8	0.0	4.2	0.0	

## Data Availability

Raw data are available upon reasonable request. All data relevant to the study are included in the article.

## Supplementary Materials

The following are available online at https://www.mdpi.com/article/10.3390/diagnostics11122351/s1, Supplementary Figure S1: Antibody validation by comparison of antibodies. The panels show a complete concordance of staining results obtained by two independent arginase-1 antibodies. Using MSVA-511R, there was a strong nuclear and cytoplasmic staining of hepatocytes (A) and weak to moderate nuclear and cytoplasmic staining of the granular layer of keratinizing squamous epithelium of the skin (B,C). Using Cell Marque clone SP156, nearly identical staining is seen in hepatocytes (D), and the skin (E,F). The images (A–C) and (D–F) were taken from consecutive tissue sections. Magnification 100×, TMA spot size 600 µm.
