# Supplementary figures and images for: Large-Scale Tissue Microarray Evaluation Corroborates High Specificity of High-Level Arginase-1 Immunostaining for Hepatocellular Carcinoma
Source: Diagnostics (Basel). 2021 Dec 14;11(12):2351. doi: 10.3390/diagnostics11122351 (PMC8699869; doi:10.3390/diagnostics11122351)

A

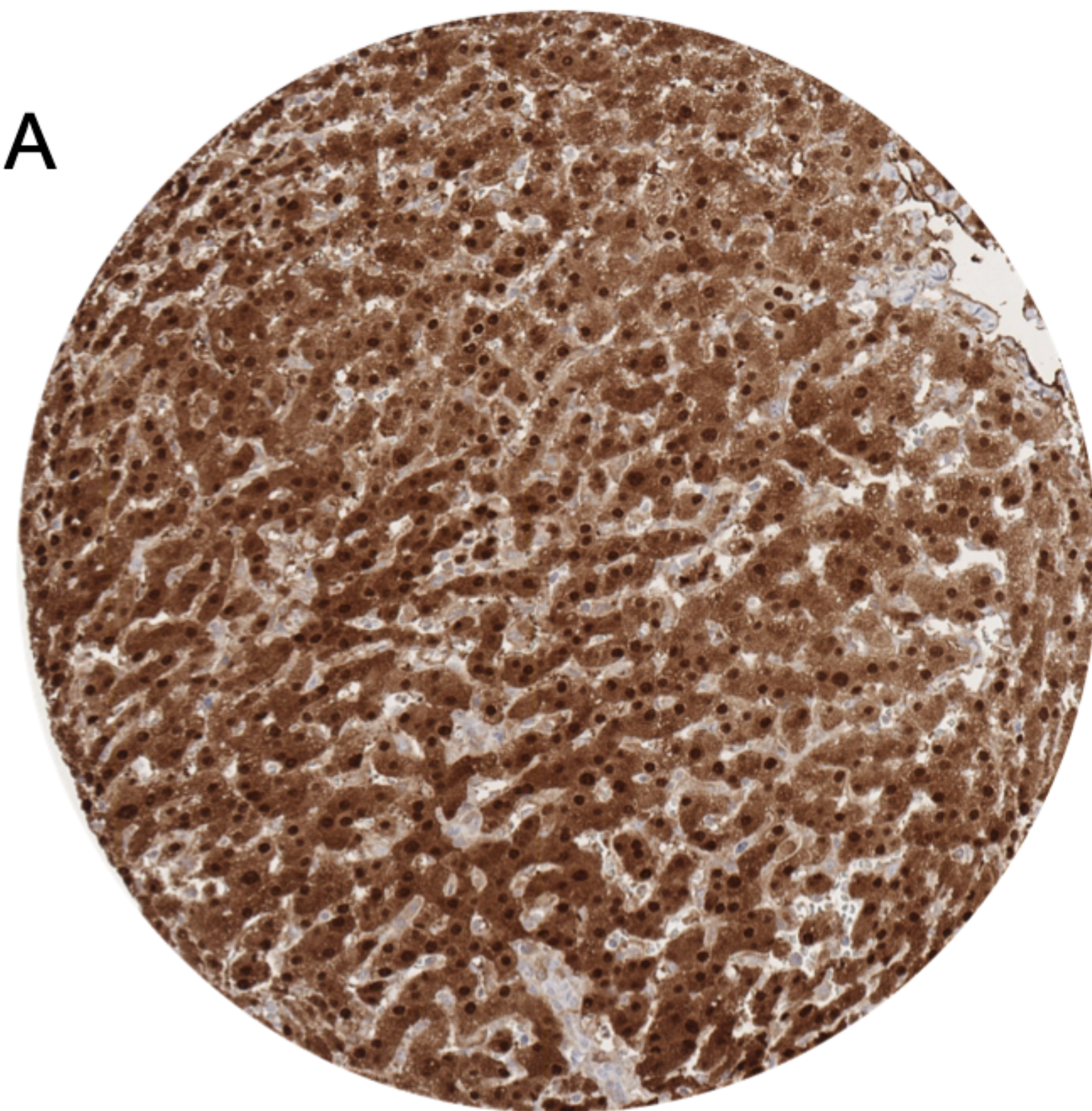

B

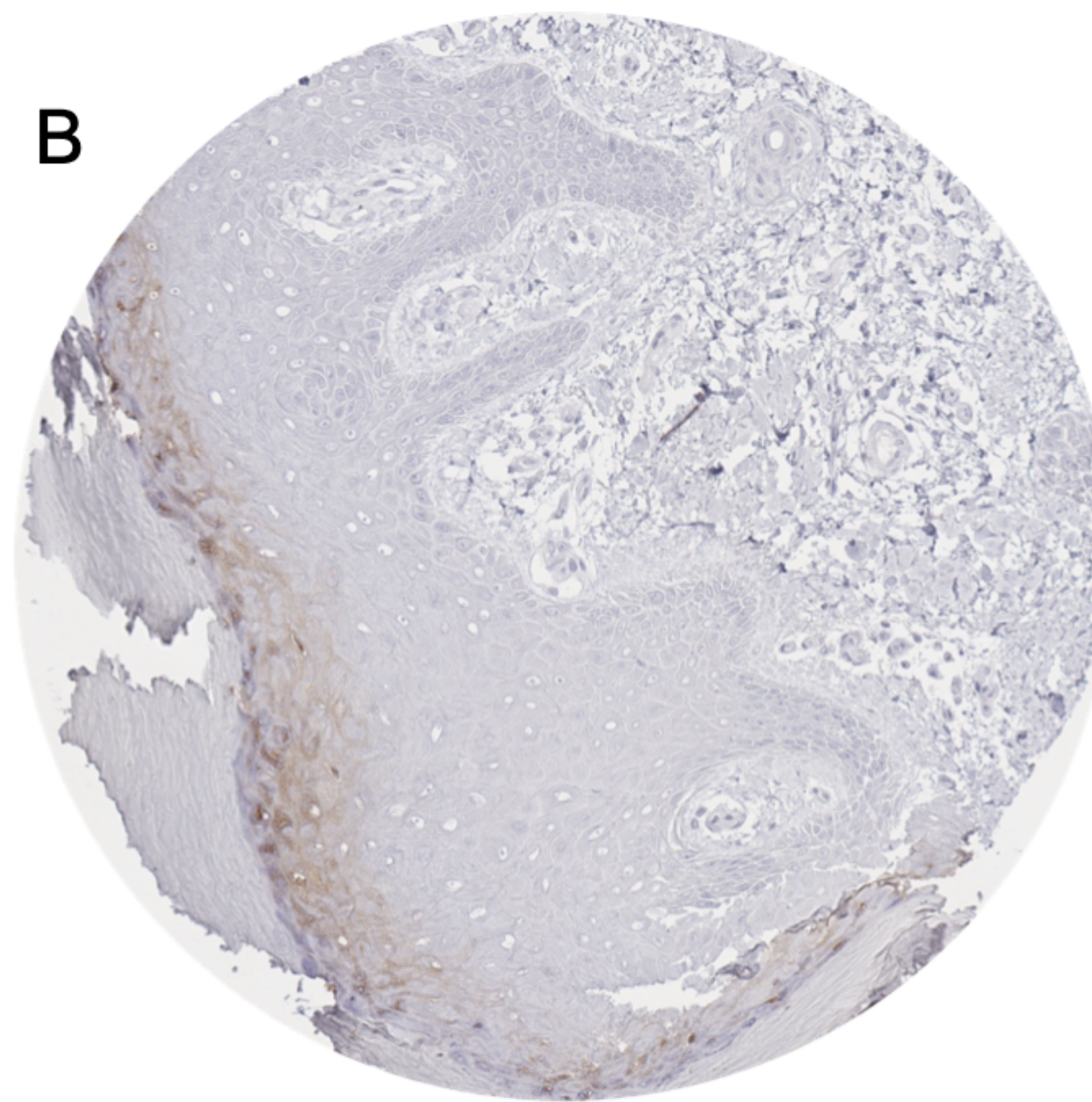

C

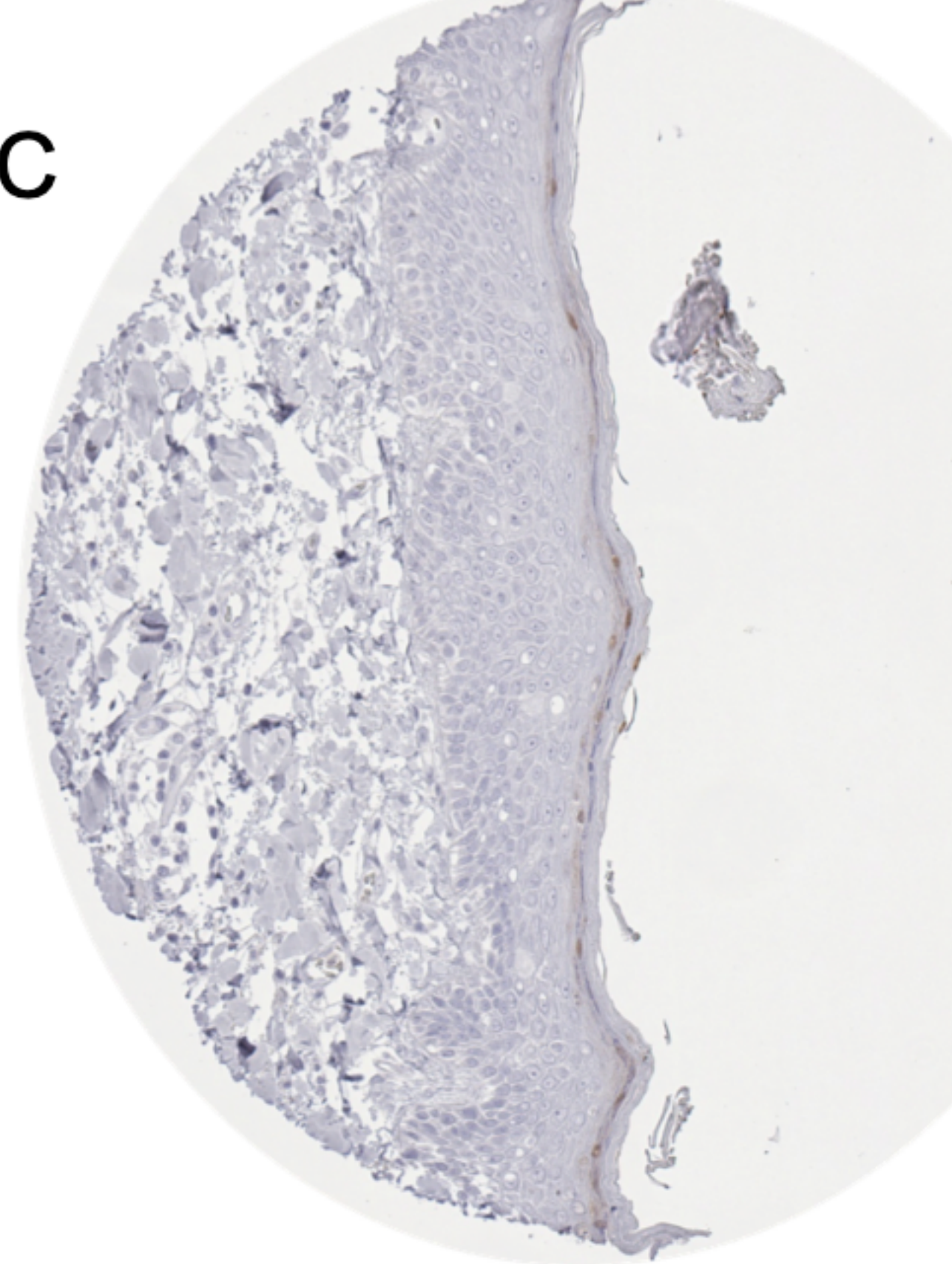

D

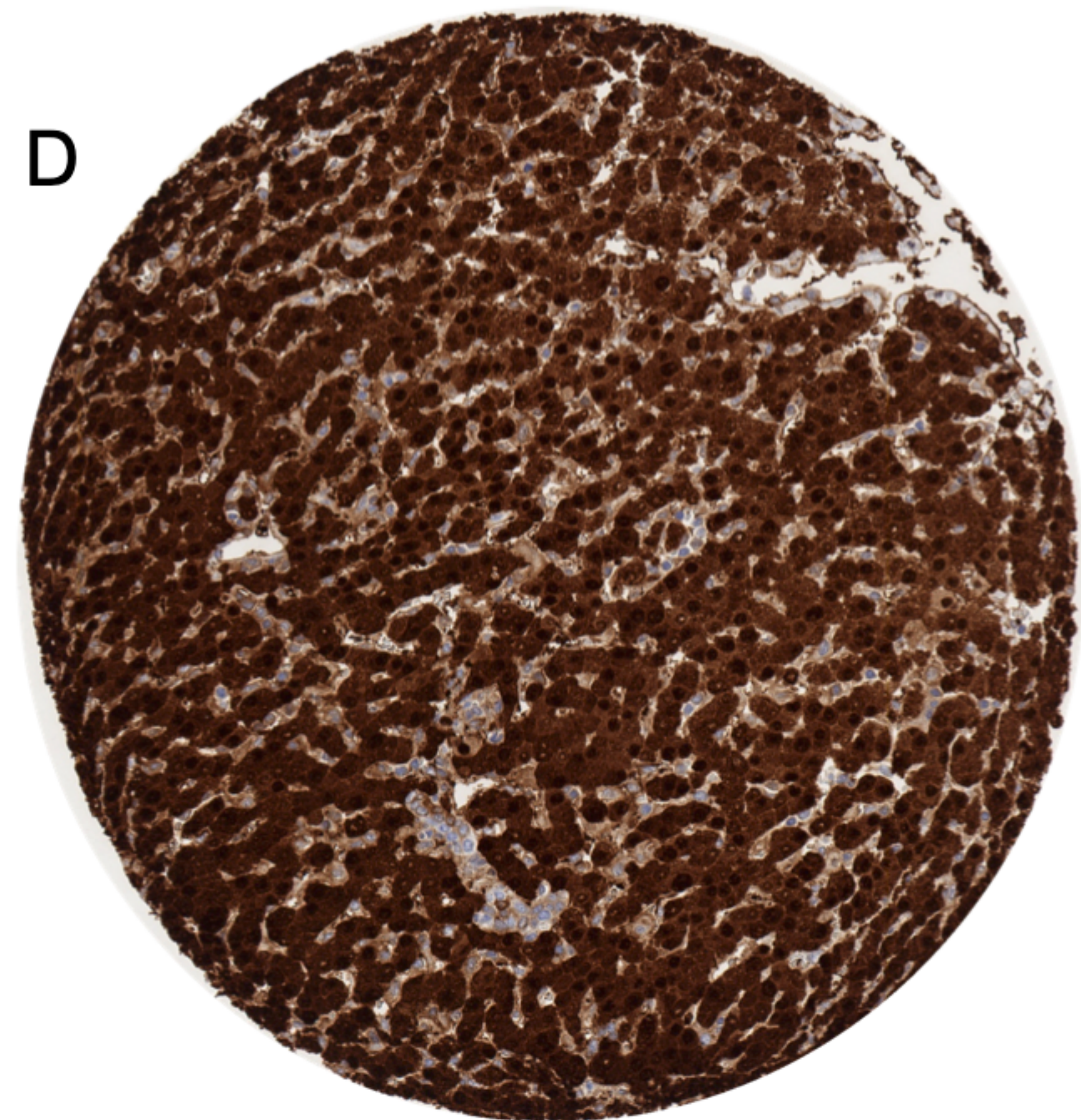

E

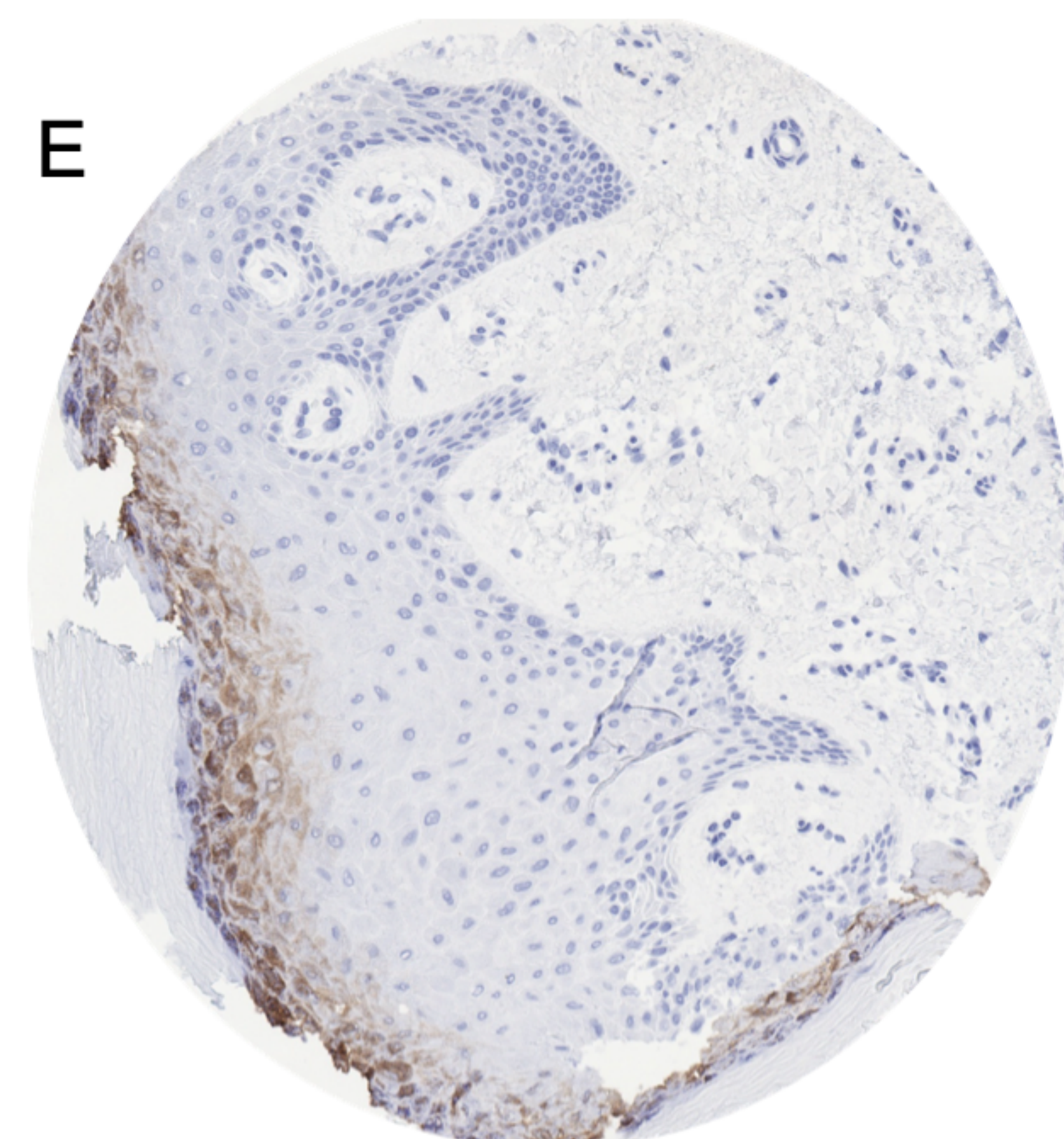

F

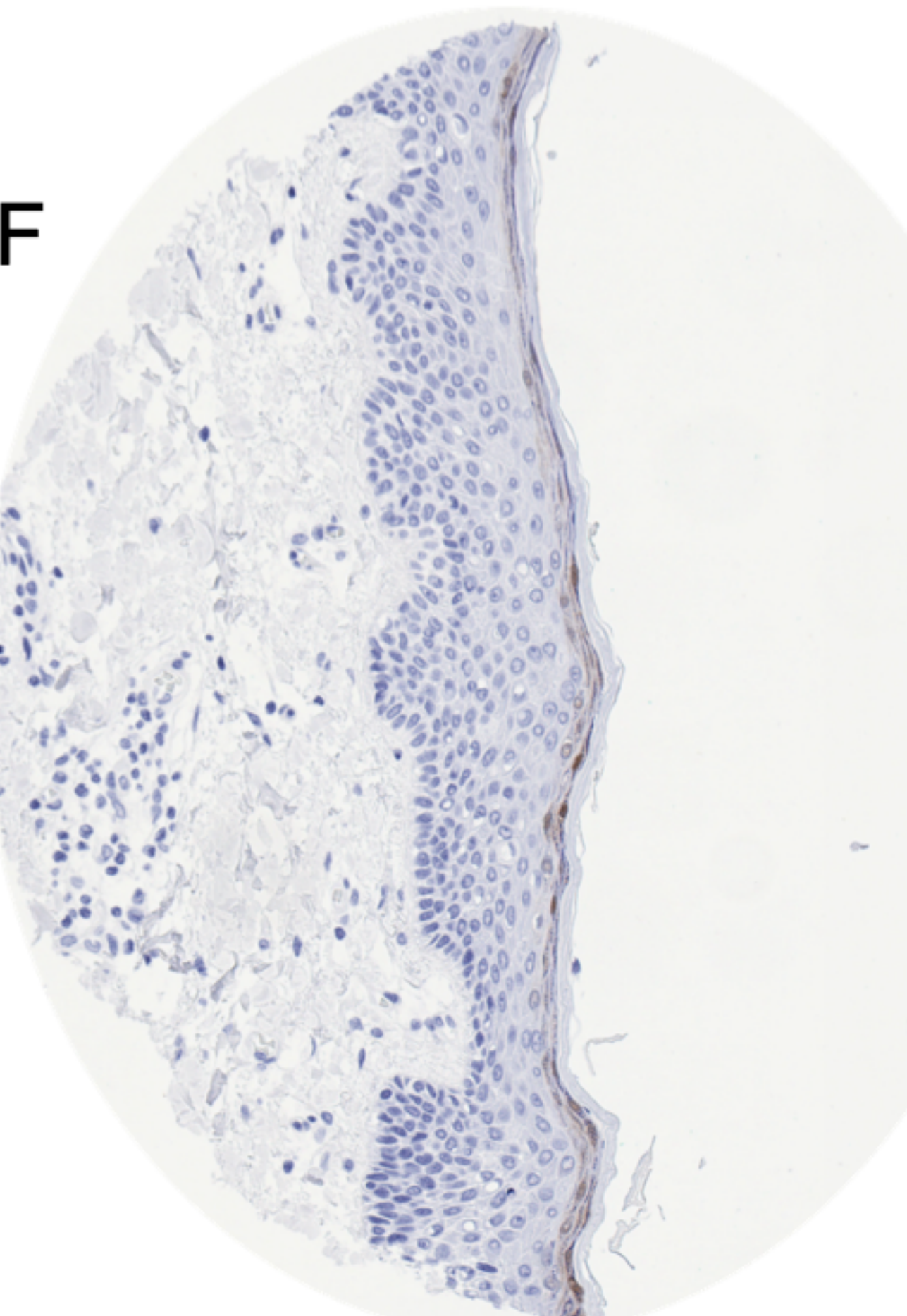

Supplement: Supplementary file 1 [file diagnostics-11-02351-s001.zip › diagnostics-1467941-supplementary.pdf]
